# Exploring the reactivity of distinct electron transfer sites at CVD grown monolayer graphene through the selective electrodeposition of MoO_2_ nanowires

**DOI:** 10.1038/s41598-019-48022-6

**Published:** 2019-09-06

**Authors:** Alejandro García-Miranda Ferrari, Christopher W. Foster, Dale A. C. Brownson, Kathryn A. Whitehead, Craig E. Banks

**Affiliations:** 1 Faculty of Science and Engineering, Manchester Metropolitan University, Chester Street, Manchester, M1 5GD UK; 2 Manchester Fuel Cell Innovation Centre, Manchester Metropolitan University, Chester Street, Manchester, M1 5GD UK; 3 Microbiology at Interfaces Group, School of Healthcare Science, Manchester Metropolitan University, Chester Street, Manchester, M1 5GD UK

**Keywords:** Electrochemistry, Physical chemistry

## Abstract

The origin of electron transfer at Chemical Vapour Deposition (CVD) grown monolayer graphene using a polymer-free transfer methodology is explored through the selective electrodeposition of Molybdenum (di)oxide (MoO_2_). The electrochemical decoration of CVD monolayer graphene with MoO_2_ is shown to originate from the edge plane like- sites/defects. Edge plane decoration of MoO_2_ nanowires upon monolayer graphene is observed *via* electrochemical deposition over short time periods only (*ca*. −0.6 V for 1 second (*vs*. Ag/AgCl)). At more electrochemically negative potentials (*ca*. −1.0 V) or longer deposition times, a large MoO_2_ film is created/deposited on the graphene sheet, originating and expanding from the original nucleation points at edge plane like- sites/defects/wrinkles. Nanowire fabrication along the edge plane like- sites/defects of graphene is confirmed with Cyclic Voltammetry, Scanning Electron Microscopy (SEM), Atomic Force Microscopy (AFM) and Raman Spectroscopy. Monitoring the electrochemical response towards [Ru(NH_3_)_6_]^**3+/2+**^ and comparing the heterogeneous electron transfer (HET) kinetics at CVD grown monolayer graphene *prior* and *post* nanowire fabrication reveals key understandings into the fundamental electrochemical properties of carbon materials. The HET kinetics (${{\boldsymbol{k}}}_{{\boldsymbol{obs}}}^{{\bf{0}}}$) at MoO_2_ nanowire decorated monolayer graphene sheets, when edge plane like- sites/defects have been coated/blocked with MoO_2_, are significantly reduced in comparison to the unmodified graphene alternative. Interestingly, MoO_2_ nucleation originates on the edge plane like- sites/defects of the graphene sheets, where the basal plane sites remain unaltered until the available edge plane like- sites/defects have been fully utilised; after which MoO_2_ deposition propagates towards and onto the basal planes, eventually covering the entire surface of the monolayer graphene surface. In such instances, there is no longer an observable electrochemical response. This work demonstrates the distinct electron transfer properties of edge and basal plane sites on CVD grown monolayer graphene, inferring favourable electrochemical reactivity at edge plane like- sites/defects and clarifying the origin of graphene electro-activity.

## Introduction

Graphene is extensively studied due to its reported unique electronic, mechanical and optical properties^1–5^, which translate into abundant research interest in energy applications such as supercapacitors^6–9^, solar cells^10–13^, fuel cells^14–16^, water splitting^17^ and for electrochemical sensors^18–20^ when utilised as an electrode material. Typically, graphene is fabricated *via* one of two routes; a bottom-up (BU) or a top-down (TD) approach. TD methods, such as chemical/thermal reduction of graphene oxide or using physical/chemical exfoliation, can give rise to a large quantity of graphene sheets; however, the fabricated graphene is generally highly defective and abundant with residual C/O groups or other contamination such as surfactants or metals^21–24^. Bottom-up (BU) fabrication routes typically lead to higher quality graphene, but in smaller quantities^25^. Chemical Vapour Deposition (CVD) growth of graphene onto a copper catalyst is a BU route that facilitates the study of high quality monolayer graphene sheets/films^26–28^, which can then either be transferred (commonly using Poly(methyl methacrylate), PMMA) onto a suitable substrate^29–31^. PMMA has previously been reported to affect the physical and electrical properties of CVD grown graphene samples transferred *via* this route^32–38^, therefore, in this work we address the fundamental properties and conduct performance studies on CVD grown monolayer graphene transferred *via* a polymer-free transfer method^39^.

In terms of understanding the electrochemical response of CVD grown monolayer graphene (*via* a BU approach), the reactivity of such electrodes is *commonly assigned* to the graphene edge planes, which are reported to exhibit *ca*. 4 orders of magnitude greater specific capacitance, faster electron transfer rates and higher electrocatalytic activity when compared to the graphene basal planes^40^. Recent work involving the electrochemical behaviour of CVD grown pristine graphene has shown a correlation in the structure of graphene, in terms of a dependence upon its number of layers and the macroscopic electrochemical response/performance^41,42^. Moreover, an identical relationship in terms of graphene’s geometric structure (the quantity of edge plane *vs*. basal plane coverage) has recently been reported with respect to the lateral flake size, in which smaller graphitic flake sizes (comprising a large edge plane like- site/defect density and a respectively small basal plane geometric contribution) exhibited improved electrochemical properties when compared to the inverse^43^. Indeed, there have been numerous other studies reporting that, in comparison to the edge plane like- sites/defects^44,45^, the basal planes of graphitic and carbon-based materials are effectively inert^40,41,46,47^. However, regardless of the vast number of recent reports concerning graphene-based electrodes, researchers still debate the *real* contributions of edge and basal plane like- sites/defects at the macroscopic scale, in future experiments researchers should clarify the experimental configuration of their studies, *ie*. macro- *vs* microscopic voltammetry^48^ with respect to their observed heterogeneous electron transfer (HET) kinetics^47,49–52^; further work is required to explore this edge *vs* basal argument for carbon surfaces^48^. It is these aforementioned research considerations that we investigate herein, through the utilisation of a MoO_2_ deposition technique to decorate CVD grown monolayer graphene (transferred using a polymer-free method) so that one can determine the *true electrochemical contributions* arising from its structure.

Molybdenum oxide(s) and other metal oxides have been shown to electrochemically nucleate specifically onto the edge plane like- sites/defects of highly oriented pyrolytic graphite (HOPG)^53–57^. Davies *et al*.^58^ reported that edge plane like- sites/defects on basal plane orientated HOPG (SPI-1 grade) are responsible for the voltammetric signal, and Rowley-Neale *et al*.^59^ extended these insights with screen-printed graphitic electrodes (SPEs) with similar results. In these approaches, the selective deposition of metals and metal oxides onto graphite is called ‘step edge decoration’^53–56^, and has been utilised to demonstrate that electrochemical reactions at edge plane graphite are “anomalously fast” in comparison to the basal plane, which is effectively inert in comparison^57,58,60^. Through the selective coating and blockage method of the edge plane like- sites/defects with MoO_2_ nanowires (that are electrochemically insulating), a reduction in the electrochemical response towards [Ru(NH_3_)_6_]^3+/2+^ ^41,47^ was reported; clearly demonstrating that the electrochemical reactivity of the HOPG is due to edge plane like- sites/defects. To the best of our knowledge, the above elegant approach has not previously been applied towards CVD grown monolayer graphene sheets (in addition to the polymer-free transfer method utilised herein) contributing to the understanding of graphene and *all akin* carbon-based electrode materials.

## Results and Discussion

Previously reported methodologies have utilised the electrochemical decoration of MoO_2_ onto HOPG and SPEs^58,59^, where MoO_2_ selectively deposits upon the available edge plane like- site/defects, allowing their electrochemical contributions to be deduced. In consideration of these approaches, herein we adapt this method towards CVD grown monolayer graphene sheets. Presented within the Experimental Section and ESI (1.1–1.4) are details of the fabrication process, optimisation and physicochemical characterisation of the CVD grown monolayer graphene sheets and their MoO_2_ decorated counterparts. Briefly, Figure S5B depicts the full Raman spectra of a MoO_2_ decorated monolayer graphene, displaying the typical monolayer graphene D (1350 cm^−1^), G (1580 cm^−1^), 2D (2700 cm^−1^) and 2D’ (3250 cm^−1^) peaks, the presence of MoO_2_ (308 cm^−1^)^61^ and the presence of the underlying Si (514 and 985 cm^−1^) wafer (which is usually not shown in the literature and is generally ignored/not-presented even though it will be observed). Additionally, AFM images were collected in order to characterise the MoO_2_ nucleation upon the edge plane like- sites/defects as depicted in Figure S7, where the length and width of the wires is 1–2 µm and 30–75 nm respectively, which corroborates with the *selective* nucleation characterised by Rowley-Neale *et al*.^59^.

**Figure 1:**
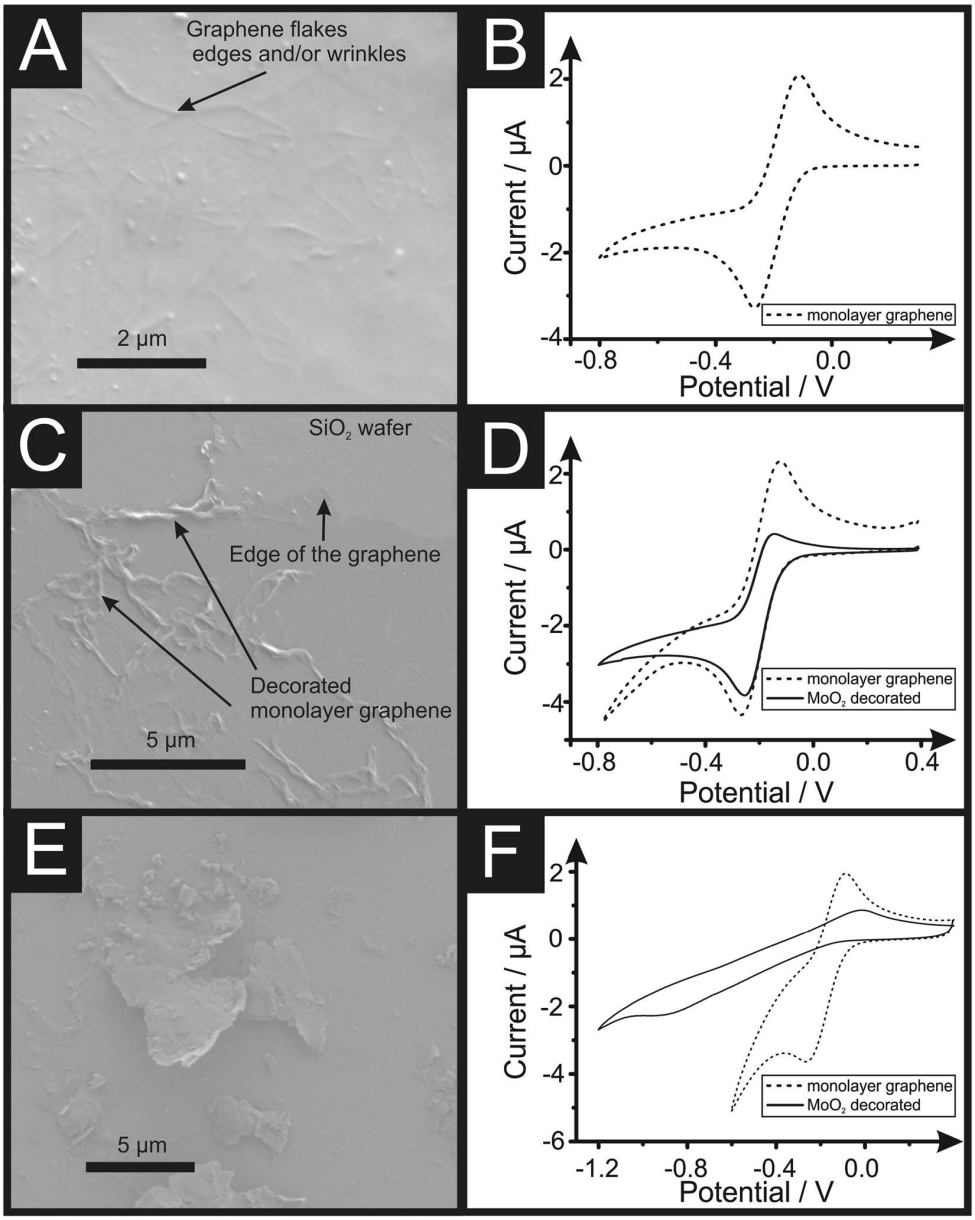
SEM images of a monolayer graphene electrode (**A**), 1 second electrodeposition of MoO_2_ at −0.6 V (vs. Ag/AgCl) (**C**) and 10 second electrodeposition of MoO_2_ at −0.6 V (vs. Ag/AgCl) (**E**). Additionally presented is cyclic voltammetric analyses recorded using 1 mM [Ru(NH_3_0_6_]^3+/2+^/0.1 M KCl, using a monolayer graphene sheet (**B**), 1 second electrodeposition of MoO_2_ at −0.6 V (vs. Ag/AgCl) (**D**) and 10 second electrodeposition of MoO_2_ at −0.6 V (vs. Ag/AgCl) (**F**) (Scan rate: 25 mV s^−1^). Note that the monolayer graphene samples (**A**) might contain dust/air impurities due to the manufacturing process explained within the Experimental Section, observed as nanoparticulates.

Figure 1A depicts an SEM image of the edge of an unmodified monolayer graphene sheet and Fig. 1B presents the corresponding cyclic voltammetric response, using the near-ideal outer-sphere redox probe, [Ru(NH_3_)_6_]^3+/2+^, which exhibits a peak-to-peak separation (*ΔE*_*p*_) of 160 mV (at 50 mV s^−1^ *vs*. Ag/AgCl) and a HET (${k}_{obs}^{0}$) rate of 1.91 × 10^−3^ cm s^−1^ (see Experimental Section). Note that this value is similar but slightly faster than other CVD monolayer graphene sheets previously reported by our research group^41^, which is due to a higher exposure of the edge plane like- sites/defects upon the graphene sheet. Furthermore, it is worth noting at this stage that each individual graphene sheet is unique in terms of its level of edge plane like- site/defects, so there is natural variation in the reported HET rates throughout the literature; thus, it is important to characterise each monolayer graphene sample^62^ prior to modification with MoO_2_. Figure 1C depicts an SEM image of the MoO_2_ electrochemically decorated monolayer graphene sheet following applying chronoamperometry at a set voltage (−0.6 V *vs*. Ag/AgCl) for 1 second, where it is readily observed that the MoO_2_ nucleates upon the edge plane like- sites/defects/wrinkles of the monolayer graphene surface. Figure 1D represents the voltammetric responses using the near-ideal redox probe, [Ru(NH_3_)_6_]^3+/2+^, which exhibits a *ΔE*_*p*_ of 200 mV (at 50 mV s^−1^ *vs*. Ag/AgCl) and a ${k}_{obs}^{0}$ of 8.80 × 10^−4^ cm s^−1^. Such decreased voltammetric behaviour implies that the insulating MoO_2_ nanowires have coated some of the available edge plane like- sites/defects, with some edge sites remaining unmodified. Figure 1D compares the voltammetric profiles and Table 1 shows (with respect to the HET kinetics (${k}_{obs}^{0}$) and the percentage of edge plane coverage (%*θ*_*edge*_) values) the changes observed as one electrodeposits MoO_2_ onto the graphene electrode’s surface. Such voltammetric profiles show that the decorated electrode (dashed line) still exhibits relatively fast HET kinetics when compared to the unmodified electrode (solid line), but presents a lower current intensity due to the limited availability of edge plane like- sites/defects on the electrode surface. This is corroborated with the SEM presented within Fig. 1C.

**Table 1:** HET kinetics (${k}_{obs}^{0}$) determined using the near ideal outer-sphere redox probe [Ru(NH_3_)_6_]^3+/2+^/0.1 M KCl, coverage of edge plane (*θ*_*edge*_) and the difference in the percentage of edge coverage upon the surface of the monolayer graphene sheets (*Δθ*_*edge*_) followed by the electrochemical decoration of MoO_2_ for: 1 (A), 2 (B), 5 (C) and 10 (D) seconds; electrochemical parameters: −0.6 V (vs. Ag/AgCl) using 1 mM Na_2_MoO_4_ (in 1 M NaCl).

Graphene Sample	Prior to electrodeposition		Electrodeposition Time (s)	Post electrodeposition		*Δ*%*θ*_*edge*_
	*k*^0^_*obs*_(cm s^−1^)	%*θ*_*edge*_		*k*^0^_*obs*_ (cm s^−1^)	%*θ*_*edge*_	
A	1.64 × 10^−3^	0.4	1	9.65 × 10^−4^	0.24	41.5
B	4.26 × 10^−4^	0.1	2	3.44 × 10^−4^	0.09	18.2
C	2.32 × 10^−3^	0.6	5	8.75 × 10^−4^	0.22	62.1
D	2.83 × 10^−3^	0.7	10	2.18 × 10^−6^	0.0005	99.9

Note: each sample (A, B, C and D) is a different CVD monolayer graphene sheet and has a varied initial size and quantity of edge sites, which exhibit distinct electron transfer rates (HET kinetics).

**Figure 2:**
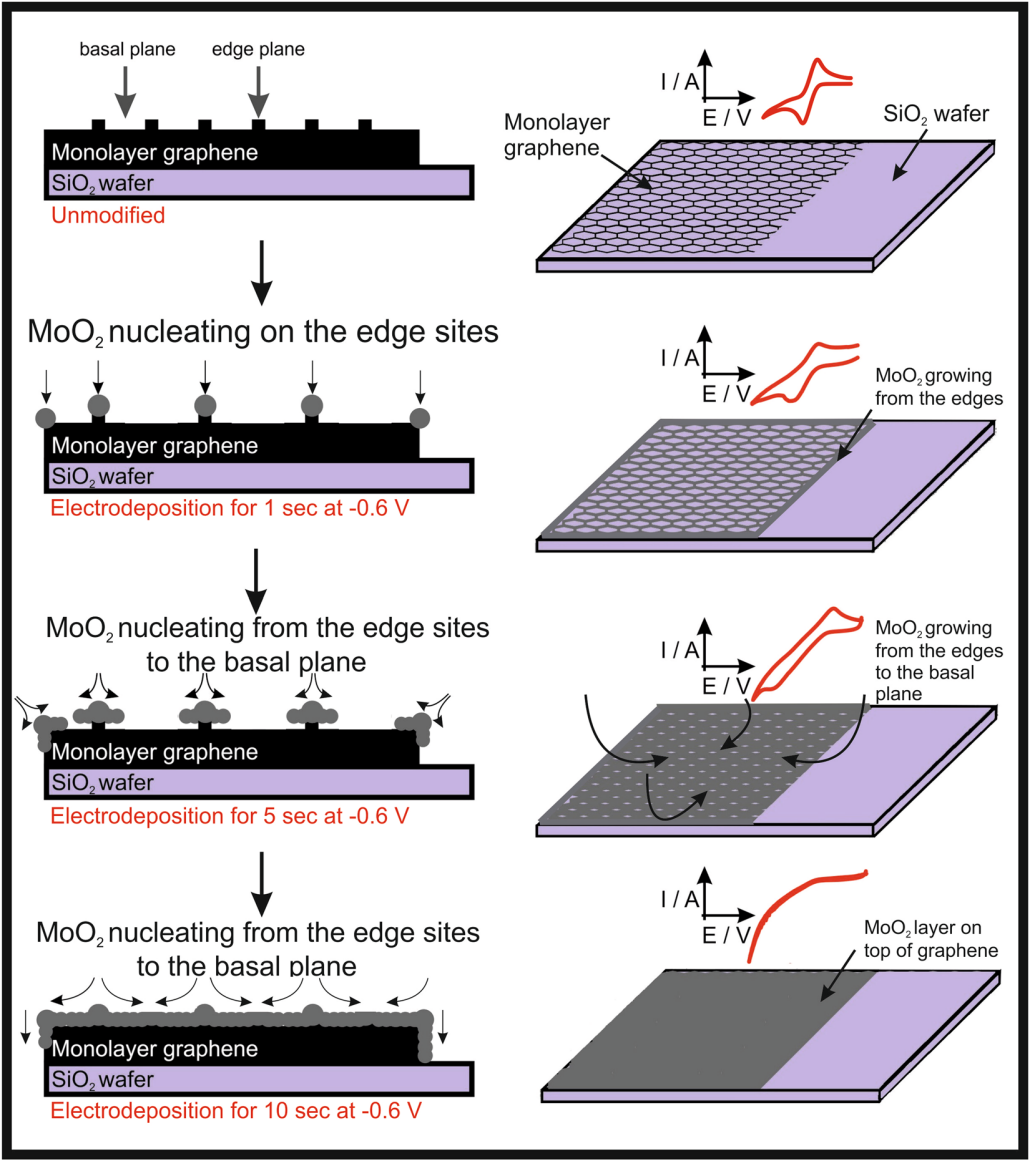
Schematic highlighting the MoO_2_ deposition process upon monolayer CVD graphene sheets, where MoO_2_ nucleation starts upon the graphene edge plane like- sites/defects. Note that longer deposition times result in the MoO_2_ growing from the edge plane like- sites/defects over onto the basal plane sites until there is a complete insulating layer covering the entire monolayer graphene sheet, resulting in substantially reduced electrochemical activity. Note, sizes are not to scale.

Next we investigate the effect of increased deposition times at −0.6 V (*vs*. Ag/AgCl). Presented within Fig. 1E is an SEM image of the MoO_2_ electrochemically decorated monolayer graphene sheet that has been applied at −0.6 V (vs. Ag/AgCl) for 10 seconds *via* chronoamperometry, where it is clear that electrodeposition is no longer isolated upon the edge plane like- sites/defects. Instead, the MoO_2_ has coated/blocked the entire electroactive surface. Upon electrodeposition at more electrochemically negative potentials (*ca*. −1.0 V) or longer deposition times, a large MoO_2_ film is created/deposited on the graphene sheet, originating and expanding from the original edge plane like- sites/defects (non-selective deposition), as shown in Figures S2 and S3. Figure 1F corroborates with this topographical analysis as the corresponding cyclic voltammetric analysis utilising the same near-ideal redox probe, [Ru(NH_3_)_6_]^3+/2+^, exhibits a *ΔE*_*p*_ of 820 mV and a ${k}_{obs}^{0}$ of 2.18 × 10^−6^ cm s^−1^. As expected, the monolayer graphene sheets coated with insulating MoO_2_ (upon the edge plane like- sites/defects), give rise to an electrochemical response with a larger *ΔE*_*p*_ (slower electron transfer) than the unmodified monolayer graphene. The aforementioned MoO_2_ coverage dependant electrochemical behaviour has been illustrated within the schematic presented within Fig. 2.

Figure 2 represents the selective nucleation process of MoO_2_ wires that nucleate onto the graphene’s edge plane (and or defect/wrinkle) sites when a electrodeposition for 1 second at −0.6 V is performed, resulting in a decrease in the reversibility of the [Ru(NH_3_)_6_]^3+/2+^ redox probe. Longer deposition times will increase the nucleation of the MoO_2_ onto remaining edge sites and towards the basal plane of the graphene sheet (deposition of 5 seconds at −0.6 V). Eventually, a complete coverage of the surface of the graphene is confirmed by the absence of cyclic voltammetric redox peaks of [Ru(NH_3_)_6_]^3+/2+^, for example, when a electrodeposition of 10 total seconds at −0.6 V is performed.

The experiments performed above confirm that the MoO_2_ wires are indeed being electrodeposited selectively onto the graphene edge plane like- sites/defects, at a set potential of −0.6 V (*vs*. Ag/AgCl) for 1 second and this results in a decrease in the ${k}_{obs}^{0}$ by 41.5% (shown in Fig. 1 and Table 1). Note that the electrochemical activity of the monolayer graphene sheet is reduced since the MoO_2_ selectively electrodeposits upon the edge plane like- sites/defects, which is confirmed by physicochemical characterisation, indicating that these are the active sites for electron transfer. If no changes in the voltammetry were observed, this would indicate that basal planes sites are the origin of electron transfer, as this is not the case, our work provides convincing evidence that it is the edge plane like- sites/defects of the CVD grown monolayer graphene that are the origin. When utilising an electrodeposition of 10 seconds, an insulating film is created, which largely decreases the amount of available edge plane like- sites/defects by up to 99.9%. Note that in the case of sample B, however, due to the initial low *θ*_*edge*_ and hence ${k}_{obs}^{0}$, in this case the deposition of MoO_2_ has fewer edge plane like- sites/defects to deposit upon. This demonstrates again that each individually grown CVD graphene sheet is unique in terms of edge plane like- sites/defects and ${k}_{obs}^{0}$; therefore emphasising the importance of their characterisation (both physicochemical and electrochemical properties of the graphene) upon fabrication and prior to application. Last, note that if incorrect deposition parameters are utilised, *i.e*. too long deposition times and high deposition potentials, physicochemical characterisation would indicate a large film is created, which would give the *false impression* that the basal plane of monolayer graphene is as *equally* electrochemically active as the edge plane sites, *which is not the case*. However, there has been a great deal of elegant work published on the micro scale response of electron transfer at carbon materials using novel nanoscale techniques^63,64^, therefore it is intended that the data reported herein be considered in-conjunction with such other studies for the electrochemical community to better understand this phenomena as a whole.

## Conclusions

MoO_2_ nanowire arrays have been electrodeposited onto the edge plane like- sites/defects of CVD grown monolayer graphene using a polymer-free transfer method, which have been confirmed *via* physicochemical characterisation. The electrochemical activity *pre* and *post* MoO_2_ electrochemical deposition has been assessed using a near-ideal redox probe and in the latter case indicates that the HET kinetics (${k}_{obs}^{0}$) are significantly reduced, providing convincing evidence that the edge plane like- sites/defects/wrinkles of the CVD grown monolayer graphene are the predominant origin of electron transfer. The confirmation of the origin of monolayer graphene’s electrochemical properties could enable its application in several areas, such as additive manufacturing, electronics, energy storage/generation and for electrochemical sensor platforms.

### Experimental section

All chemicals used were of analytical grade and were used as received from the supplier (Sigma-Aldrich, Irvine, UK) without any further purification. All solutions were prepared with deionised water of resistivity no less than 18.2 MΩ cm and were vigorously degassed prior to electrochemical measurements with high purity, oxygen free nitrogen. Test solutions were: 1 mM [Ru(NH_3_)_6_]^3+/2+^ (in 0.1 M KCl), 0.5 M H_2_SO_4_ and 1 mM Na_2_MoO_4_ (in 1 M NaCl).

Electrochemical measurements were carried out using an Autolab PGSTAT204 potentiostat (Metrohm Autolab, Utrecht, The Netherlands). All measurements were conducted using a three-electrode system. Working electrodes were in-house fabricated CVD synthesised monolayer graphene films supported on an oxidised silicon wafer. A nickel wire counter/auxiliary electrode and a silver/silver chloride electrode (Ag/AgCl) reference electrode completed the circuit.

The in-house graphene growth took place within a nanoCVD-8G rig (Moorfield, UK), which uses a cold-wall variant of the CVD process, using a method based on that reported previously by Bointon^65^. Copper foils (25 µm, 99.99 + %) were placed on the heating stage (1 × 1 cm^2^ sample) and sealed within the vacuum chamber. The CVD system is then heated from room temperature to 1035 °C (growth temperature) in 3 minutes with H_2_ gas flowing at a constant rate of 0.4 sccm at 1.33 Pa. Following this, the annealing step was performed for 300 seconds at 1035 °C in the constant H_2_ atmosphere. Next, a nucleation and growth process is initiated at 1000 °C, with the first step lasting 120 seconds (with a flow of 1.4 sccm of CH_4_ and 0.4 sccm of H_2_ and a pressure of 1.33 Pa) followed by the second step (growth) for 720 seconds (at 7 sccm of CH_4_ and 0.4 sccm of H_2_ kept constant flow rate and a pressure of 1.33 Pa). Next, there is a cooling down process, based on a flow of H_2_ at 0.4 sccm and Ar at 100 sccm, which reduces the temperature from 1000 °C to 190 °C for 600 seconds at 1.33 Pa. Finally, the sample is left to cool at atmospheric pressure and with no gas flow until a temperature below 40 °C is reached to avoid any copper/sample oxidation. A modified version of a polymer free transfer method is utilised in this work, adapted from Zhang^39^, which makes use of an organic/aqueous biphasic configuration made from hexane/ammonium persulfate; where the ammonium persulfate acts as an etching solution to remove the copper and in doing so avoids the use of polymers that can contaminate the fabricated graphene^30,34^. After etching of the supporting copper, the graphene sheets are transferred into a deionised water/hexane interface to clean away any etching product that might still be in solution (this process is repeated several times). Finally, the free-floating graphene sheet is transferred onto a Si/SiO_2_ wafer to be used as an electrode for further studies.

SolidWorks software has been used to design the 3D printed electrochemical cell, which has been printed using an ‘ultraviolet curable proprietary polymer’ and a “Form 2 3D printer” (from Formlabs, USA). The CVD grown graphene working electrode was secured into the 3D printed electrochemical cell described in Figure S8 and was ‘connected to’ with copper foil to a crocodile connector, which leads to the potentiostat and external reference and counter electrodes.

The HET rate constants, ${k}_{obs}^{0}$, were calculated using the near ideal outer-sphere redox probe [Ru(NH_3_)_6_]^3+/2+^ (in 0.1 M KCl) using the well-known^66^ and utilised Nicholson method^67^, for *quasi*-reversible electrochemical reactions *via* the following formula^59^:

1 $$\phi ={k}_{obs}^{0}{[\pi Dn\nu F/RT]}^{-1/2}$$

where *φ* is a kinetic parameter, *D* is the diffusion coefficient for [Ru(NH_3_)_6_]^3+/2+^ (*D* = 9.1 × 10^−6^ cm^2^ s^−1^)^66^, *n* is the number of electrons that are taking part in the process, *F* is the faraday constant, *v*is the scan rate, *R* is the gas constant and *T* is the temperature in Kelvin. In order to calculate the HET rate constant, we use the peak to peak separation (*ΔE*_*p*_) to deduce *φ*, where ΔE_p_ is obtained at various voltammetric scan rates^52^. The standard heterogeneous constant (${k}_{obs}^{0}$) can be calculated *via* the gradient when plotting *φ* against *[πDn vF/RT]*^−*1/2*^. In cases where ΔE_p_ is bigger than 212 mV, the following equation should be implemented:

2 $${k}_{obs}^{0}=[2.18{(\frac{{\rm{\alpha }}Dn\nu F}{RT})}^{-\frac{1}{2}}\exp \,[-(\frac{{\rm{\alpha }}\mathrm{nF}}{RT})\Delta \mathrm{Ep}]$$

where α is assumed to be 0.5.

The observed electron transfer rate, ${k}_{obs}^{0}$, of graphite electrodes has been shown to be a contribution of edge plane like- sites/defects (${k}_{edge}^{0}$) and basal planes (${k}_{basal}^{0}$), allowing one to calculate the specific contributions with the following equation:

3 $${k}_{obs}^{0}={k}_{edge}^{0}({{\rm{\theta }}}_{edge})+{k}_{basal}^{0}(1-{{\rm{\theta }}}_{edge})$$

where ${{\rm{\theta }}}_{edge}$ is the coverage of edge plane like- sites/defects on the surface of the electrode, and ${k}_{edge}^{0}$ has been shown as anomalously fast over that of ${k}_{basal}^{0}$ on graphite, allowing one to write:

4 $${k}_{edge}^{0}\gg {k}_{basal}^{0}$$

Such that Equation (3) now becomes:

5 $${k}_{obs}^{0}={k}_{edge}^{0}({{\rm{\theta }}}_{edge})$$

allowing the coverage of edge plane like- sites/defects upon the graphite electrode to be deduce; we use this and adapt this to the voltammetry observed at the CVD grown monolayer graphene sheets. %θ_*edge*_ is the percentage representation of the edge plane coverage in comparison to the total coverage, being the sum of %θ_*edge*_ and %θ_*basal*_ always equalling 100%. In the above analysis, the assumption is that the electron transfer rate for edge plane like- sites/defects (${k}_{edge}^{0}$) for the near ideal outer-sphere redox probe, [Ru(NH_3_)_6_]^3+/2+^, has been widely determined with a commercial simulation package providing a value of 0.4 cm s^−1^ for HOPG electrodes^58^.

Raman Spectroscopy was performed using a DXR Raman Microscope (Thermo Scientific, UK) fitted with a 532 nm excitation laser at a low power of 3 mW to avoid any heating effects. Spectra were recorded using a 3 second exposure time for 3 accumulations at each point. Scanning electron microscope (SEM) images were obtained using a JSM-5600LV (JEOL, Japan) model SEM equipped with an energy-dispersive X-ray microanalysis (EDS) package. Atomic force microscopy (AFM) images were obtained using a DualScope C26 (DME, Germany), carried out using AC mode using DS 95 AC probes with a spring constant of 42 N m^−1^ (DME, Germany). Scans were carried out at 40 µm/s with a force set point of 6 nN. Samples were attached to glass microscope slides using double sided tape.

Note that the area studied is in the macroscale range which is not considered too large because indeed it averages the actual phenomena occurring locally (which we study in terms of physicochemical characterisation on the nanoscale too). Such that this makes this study applicable for real applications. We are reporting on the average response of the macro electrode with varying contributions of the two micro features (edge and basal planes), which is relevant because the likely end use/application of such systems will be on that of the macro scale.

## Supplementary information

Supplementary information
