# Supplementary material for: Exploring the reactivity of distinct electron transfer sites at CVD grown monolayer graphene through the selective electrodeposition of MoO2 nanowires
Source: Sci Rep. 2019 Sep 6;9:12814. doi: 10.1038/s41598-019-48022-6 (PMC6731215; doi:10.1038/s41598-019-48022-6)
Supplement: Supplementary file 1 — Supplementary information [file 41598_2019_48022_MOESM1_ESM.pdf]

Electronic Supporting Information (ESI)

Exploring the reactivity of distinct electron transfer sites at CVD grown monolayer graphene through the selective electrodeposition of MoO_2 nanowires

Alejandro García-Miranda Ferrari [a,b], Christopher W. Foster [a,b],

Dale A. C. Brownson [a,b]*, Kathryn A. Whitehead [c], and Craig E. Banks [a,b]*

[a]: *Faculty of Science and Engineering, Manchester Metropolitan University, Chester Street, Manchester M1 5GD, UK.*

[b]: *Manchester Fuel Cell Innovation Centre, Manchester Metropolitan University, Chester Street, Manchester M1 5GD, UK.*

[c]: *Microbiology at Interfaces Group, School of Healthcare Science, Manchester Metropolitan University, Chester Street, Manchester M1 5GD, UK.*

**To whom correspondence should be addressed.*

C.E.B. / Email: c.banks@mmu.ac.uk; Tel: ++(0)1612471196; Fax: ++(0)1612476831;
Website: www.craigbanksresearch.com

D.A.C.B. / Email: d.brownson@mmu.ac.uk; Tel: ++(0)1612476561

1.1 Optimising the electrochemical deposition of MoO_2 upon the monolayer graphene sheets

A monolayer graphene sheet covering half of the SiO_2 wafer surface, was used to highlight the edge plane sites/defects of the monolayer graphene, creating a single step of one carbon atom as depicted in Figure S1. The monolayer graphene electrode was immersed in solutions of 0.5 and 1 mM Na_2MoO_4 (in 1 M NaCl and 1M NH_4Cl adjusted to pH 8.5 with liquid NH_3). Linear sweep voltammetry was performed from 0.5 to -1.5 V (*vs.* Ag/AgCl) as depicted in Figure S4, where the electrochemical deposition of MoO_2 onto the electrode surface is detected *via* the electrochemical reduction peak at -0.6 V (*vs.* Ag/AgCl). In this electrochemical process, the reduction of Mo^{6+} to Mo^{4+} occurs through the following reaction mechanism: $MoO_4^{2-} + 2H_2O + 2e^- \rightarrow MoO_2 + 4OH^-$, producing MoO_2 deposited on the monolayer graphene. Note that in the experiments performed herein, the potential at which the MoO_2 electrodeposition occurs is shifted to a less negative potential compared to -1.0 V as reported previously when using HOPG[1,2] and graphitic SPEs[3], which is likely due to the use of inert binders in the SPEs, and that we have exposed the edge plane of the monolayer graphene making it more readily available. Inspection of the voltammetric signals shown in Figure S4 reveal deposition potentials of -0.6 V (*vs.* Ag/AgCl), which corresponds to the onset of MoO_2 deposition, and resultantly the required chronoamperometry was subsequently performed at this potential.

Figure S1. Optical (A) and SEM (B) images of a monolayer graphene sheet deposited upon a SiO_2 wafer covering half of surface in order to expose the edge plane sites/defects of the monolayer graphene before going into the electrochemical cell. Note that the graphene samples may possess air/dust impurities in the form of dots apparent in B.

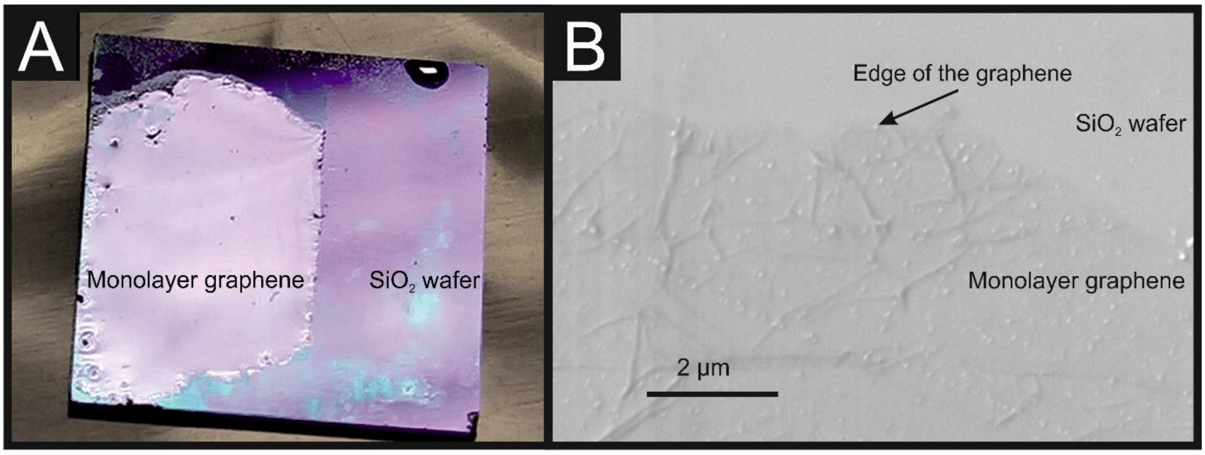

Figure S2. SEM images of a monolayer graphene sheet decorated with MoO_2 through electrodeposition at – 0.6 V (vs. Ag/AgCl, Scan rate: 5mV s^{-1}) for 1 (A), 5 (B), 20 (C) and 100 seconds (D) respectively. Images show how with longer deposition times, deposition is no longer edge plane selective and moves from the edge to the basal planes, covering the entire graphene sheet.

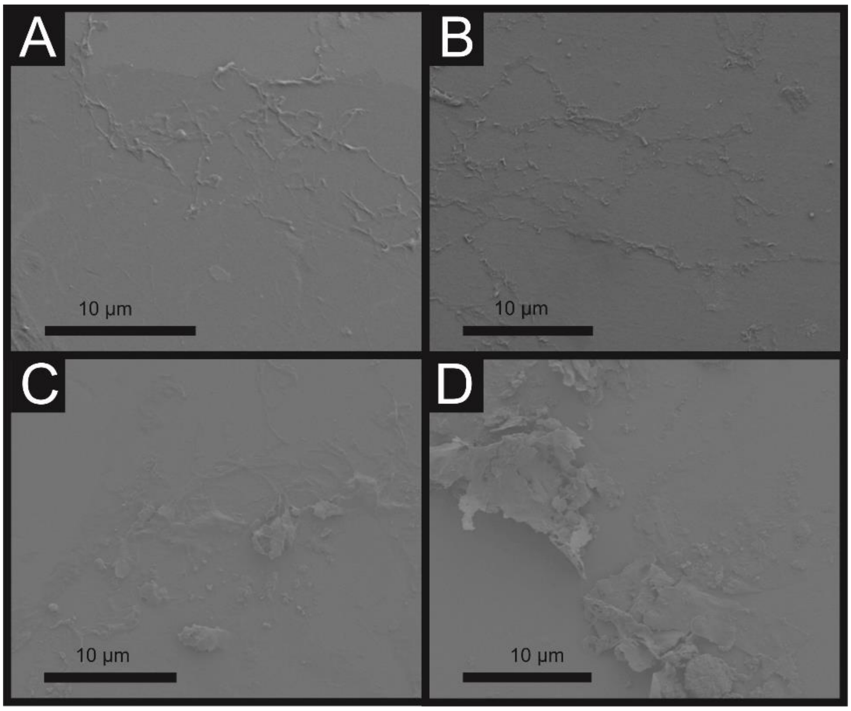

Figure S3. SEM images of a monolayer graphene sheet decorated with MoO_2 through electrodeposition at – 1 V (vs. Ag/AgCl, Scan rate: 5mV s^{-1}) for 1 (A), 5 (B), 20 (C), 100 (D) and 200 seconds (E) respectively. Images show how with a more negative potential and longer deposition times, deposition is no longer edge plane selective (when is MoO_2 nanowires) and moves from the edge to the basal planes (non-selective deposition, as MoO_2 bulk), covering and breaking the entire graphene sheet.

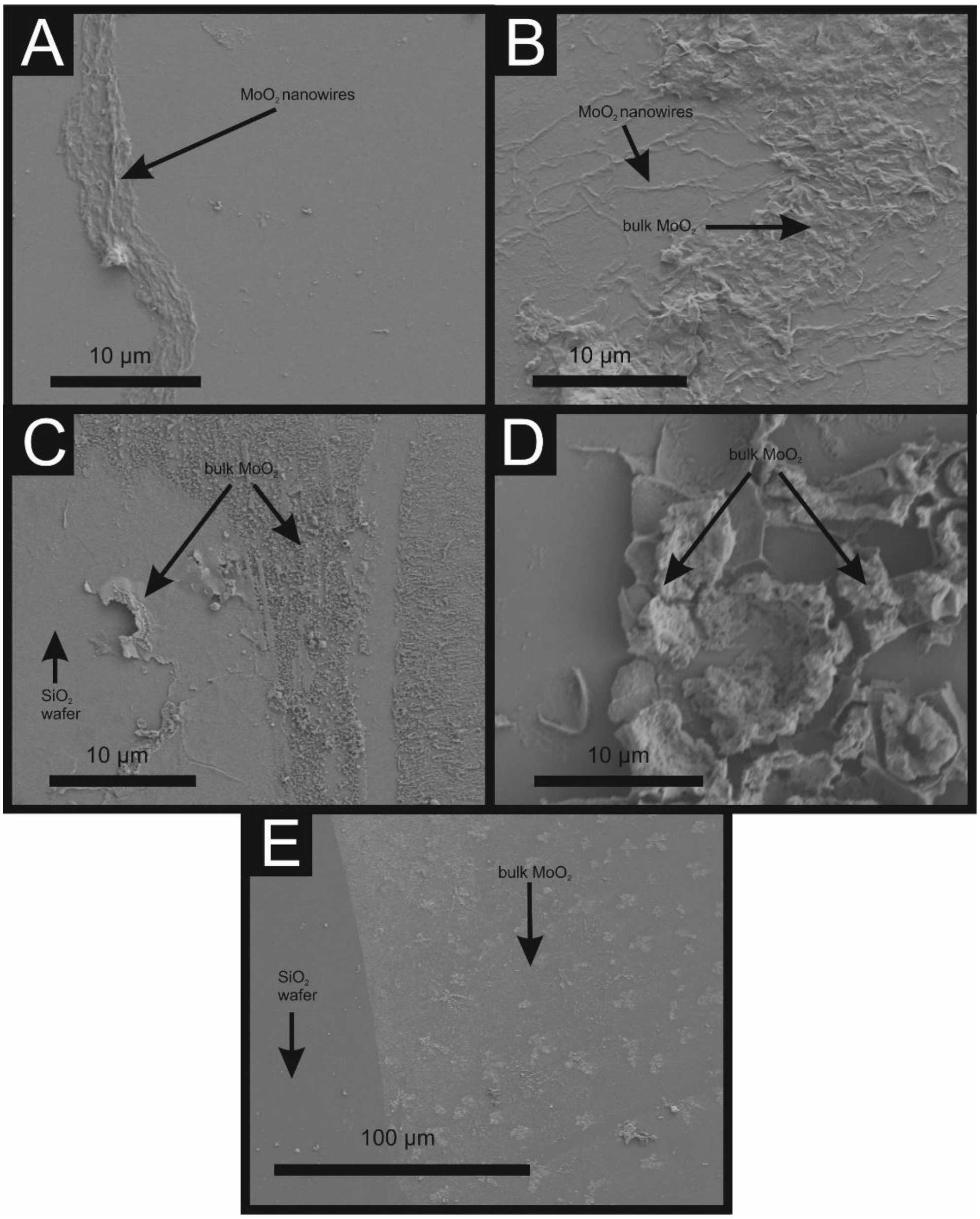

Figure S4. Linear sweep voltammetry recorded with 0.5 (solid line) and 1 mM (dotted line) Na_2MoO_4 solutions depicting the electrodeposition of MoO_2 at a monolayer graphene sheet (vs. Ag/AgCl; Scan rate 5 mV s^{-1}).

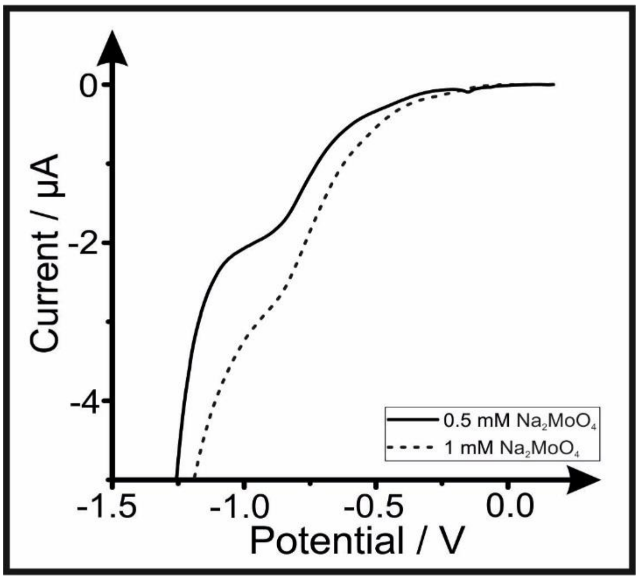

1.2 Raman characterisation

Raman characterisation of the MoO_2 decorated monolayer graphene is recorded over the range: 20–3300 cm^{-1}. The schematic presented in Figure S5A depicts the electrodeposition process of MoO_2 nucleating onto the monolayer graphene edge plane like- sites/defects, when the electrochemical decoration is held -0.6 V (*vs.* Ag/AgCl) for 1 second. Figure S5B shows the full Raman spectra of a MoO_2 decorated monolayer graphene, displaying the typical monolayer graphene D (1350 cm^{-1}), G (1580 cm^{-1}), 2D (2700 cm^{-1}) and 2D' (3250 cm^{-1}) peaks, the presence of MoO_2 (308 cm^{-1})[4] and the presence of the Si (514 and 985 cm^{-1}) wafer (which usually are not shown in the literature). Figure S5C depicts the Raman analysis of an area where MoO_2 has not grown yet, indicating the presence of monolayer graphene and the Si wafer. Figure S5D shows the Raman spectra of an area of the chip where there is no monolayer graphene and therefore there is no presence of MoO_2, showing only the presence of the Si wafer.

Figure S5. Schematic of selective MoO_2 deposition process (chronoamperometry at -0.6 V (*vs.* Ag/AgCl) for 1 second), where the monolayer graphene sheet covers half of the SiO_2 wafer (A). Raman spectra from the edge of the monolayer graphene (B) with a MoO_2 peak at 308 cm^{-1}, the monolayer graphene where MoO_2 has not been electrodeposited yet (C), and an area where there is only SiO_2 wafer (D).

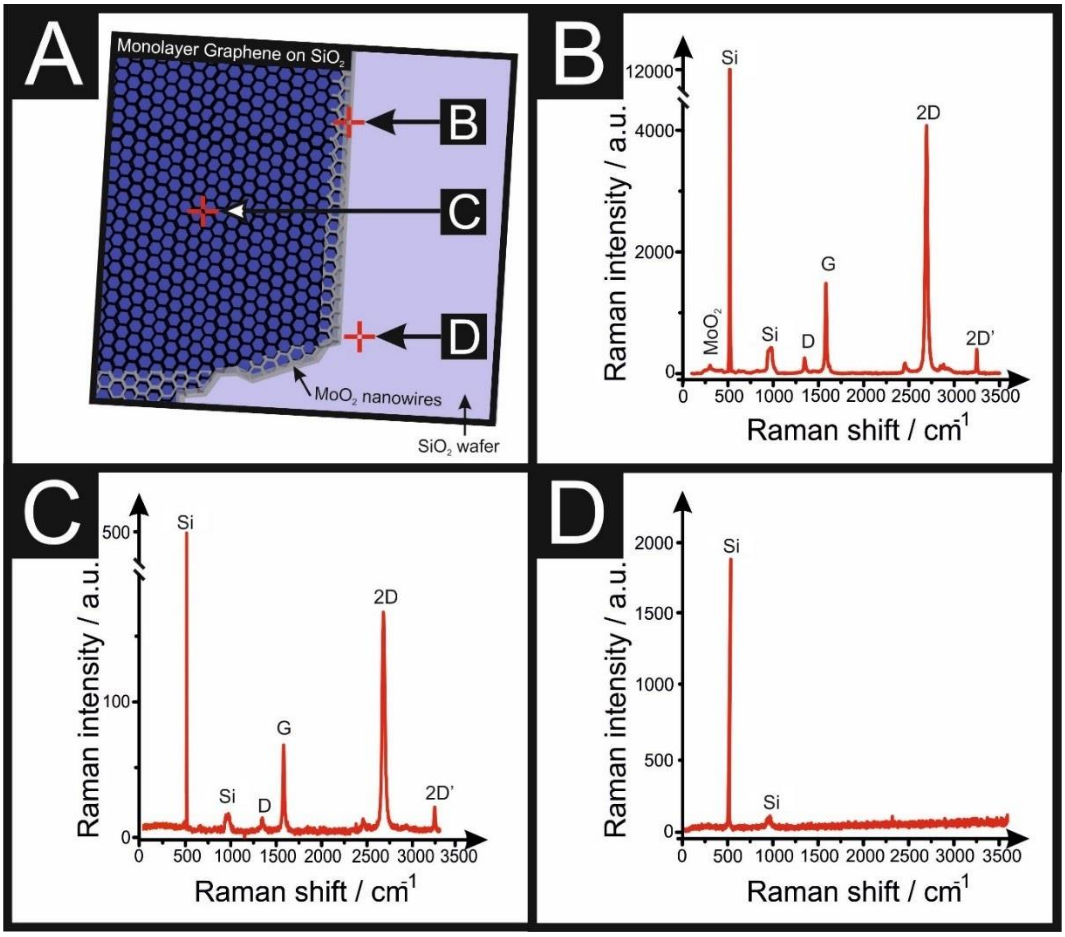

Figure S6. Raman spectra from the edge of the monolayer graphene before (A) and after (B) its decoration with MoO_2 (chronoamperometry at -0.6 V (*vs.* Ag/AgCl) for 1 second). Raman peak at 308 cm^{-1} corresponds to the MoO_2 nanowires on the decorated graphene electrode.

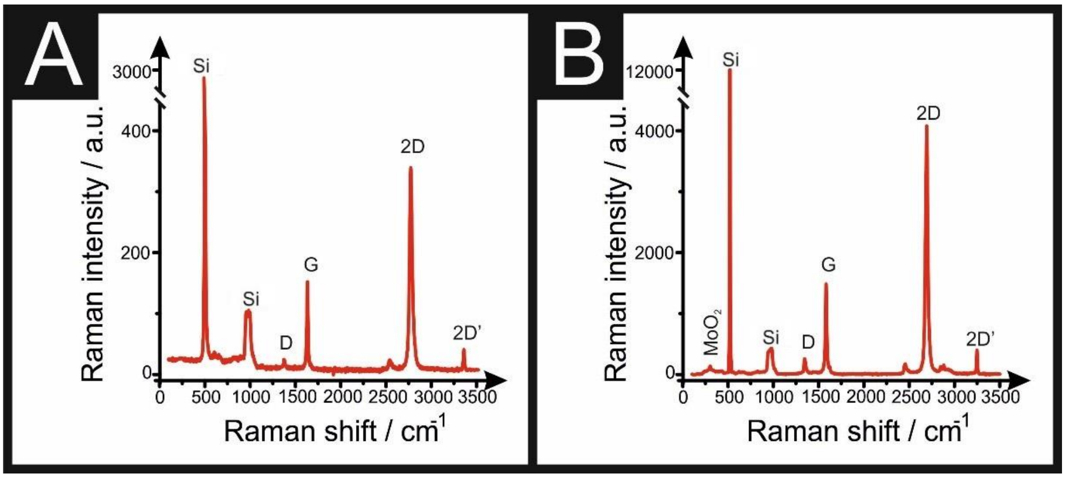

1.3 AFM characterisation of the selective electrodeposition of MoO_2 upon a monolayer graphene sheet

AFM images were collected in order to characterise the MoO_2 nucleation upon the edge plane like- sites/defects as depicted in Figure S7, where the length and width of the wires is 1-2 µm and 30-75 nm respectively, which corroborates with the *selective* nucleation characterised by Rowley-Neale *et al.*[3].

Figure S7. AFM analysis of a monolayer graphene sheet following the electrodeposition of MoO_2 at -0.6 V for 1 second (*vs.* Ag/AgCl). Figure A shows the topography of the graphene, where most of the surface remains unmodified (basal plane), although some MoO_2 wires can be observed in B, C, D and E.

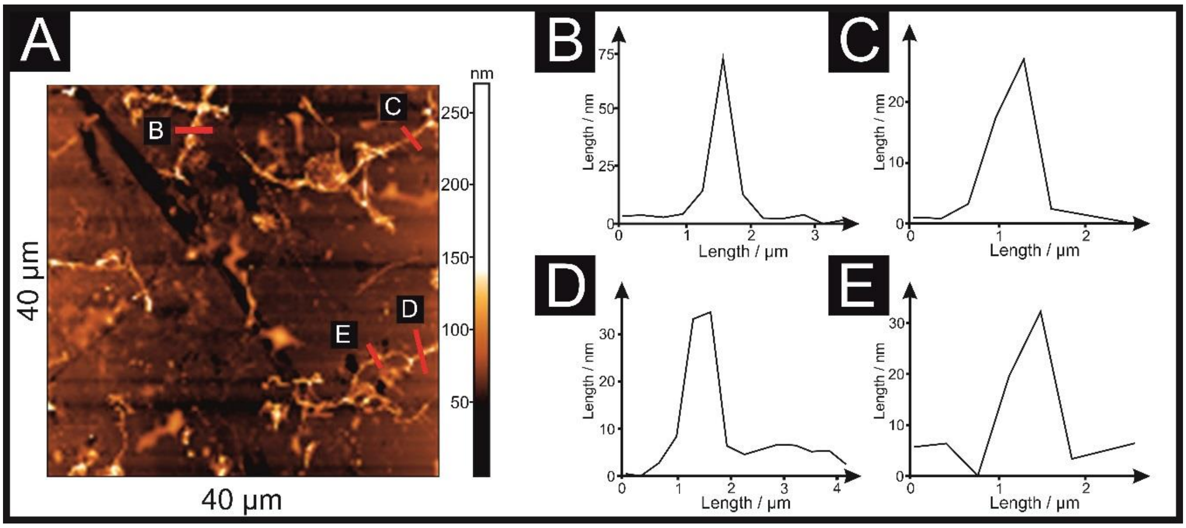

1.4 3D printed electrochemical cell to facilitate experiments/testing of the monolayer graphene sheets

Figure S8. Optical image of the CVD graphene 'housing' 3D printed cell (A) designed using Solidworks and 3D printed using a Form 2 3D UV curable printer. Schematic perspective (B) and cross-sectional view (C) of the CVD grown monolayer graphene sheet when reference and counter electrodes are incorporated into the three-electrode system in the 3D printed cell. Schematic diagram of the CVD graphene 'housing' 3D printed cell (D). The cell allows the connection of the monolayer graphene sheet to the potentiostat with a copper foil wrapping the graphene chip and allowing its use under a microscope or Raman Spectrometer without dismounting and manipulating the chip. There is a silicon O-ring sealing the graphene chip, keeping the studied area of the WE constant. There is extra space to contain the liquid solution and the external reference and counter electrodes.

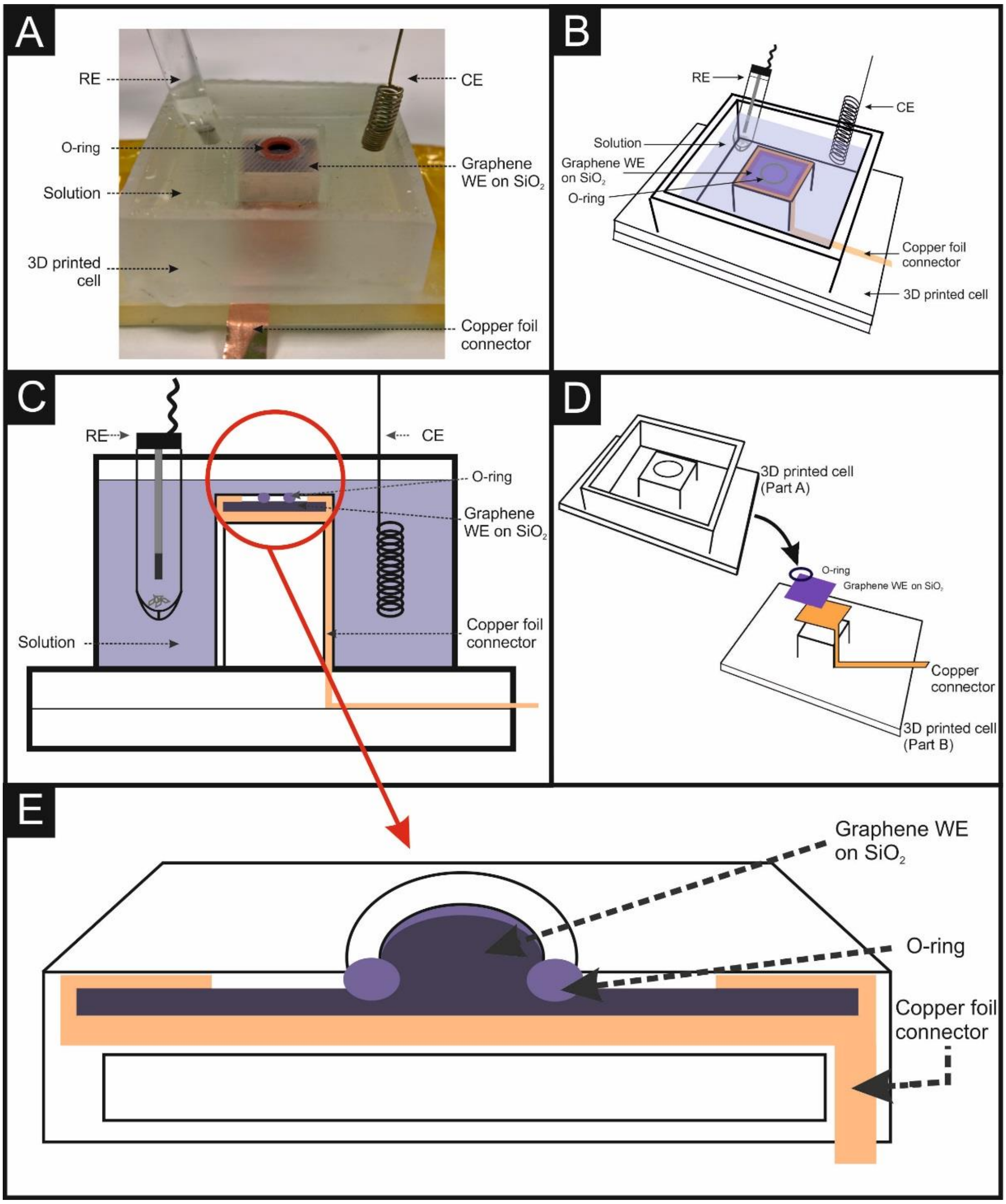

References

1. Walter, E. C. *et al.* Metal Nanowire Arrays by Electrodeposition. ChemPhysChem **4** (2), 131-138 (2003).

2. Davies, T. J.; Hyde, M. E.; Compton, R. G., Nanotrench arrays reveal insight into graphite electrochemistry. Angew Chem **44** (32), 5121-6 (2005).

3. Rowley-Neale, S. J.; Brownson, D. A. C.; Banks, C. E., Defining the origins of electron transfer at screen-printed graphene-like and graphite electrodes: MoO2 nanowire fabrication on edge plane sites reveals electrochemical insights. Nanoscale **8** (33), 15241-15251 (2016).

4. Camacho-López, M. A *et al.*, Micro-Raman study of the m-MoO2 to α-MoO3 transformation induced by cw-laser irradiation. Optical Materials **33** (3), 480-484 (2011).
